# Adjustment Disorders and Quality of Life in Patients With Tracheostomy and Tracheal Cannula: A Scoping Review

**DOI:** 10.1002/brb3.70517

**Published:** 2025-05-27

**Authors:** Andrea Calderone, Francesco Corallo, Davide Cardile, Carmela Rifici, Fabrizia Caminiti, Valeria Conti‐Nibali, Serena Filoni, Rocco Salvatore Calabrò

**Affiliations:** ^1^ Department of Clinical and Experimental Medicine University of Messina Messina Italy; ^2^ IRCCS Centro Neurolesi Bonino‐Pulejo Messina Italy; ^3^ Unit of Neuro‐Rehabilitation IRCCS Casa Sollievo della Sofferenza San Giovanni Rotondo Italy

**Keywords:** anxiety, depression, quality of life, rehabilitation, stress, tracheostomy

## Abstract

**Introduction:**

Tracheostomy (TCT) surgery and subsequent tracheal cannula (TC) insertion involve several physical, communicative, and psychological changes for the patient. Decreased mood and dysfunctional states such as morbid anxiety, depression, and stress are often experienced in these clinical situations, and affect the quality of life (QoL) and the rehabilitation process. This review aims to examine the studies in the literature on adjustment disorders (AjD) in patients with TCT or TC and how this affects their QoL during the rehabilitation process.

**Methods:**

Studies were identified from an online search of PubMed, Cochrane Library, and Embase databases. Studies published between 2013–2023 were selected. This review has been registered on OSF (n): DOI 10.17605/OSF.IO/RHX3J.

**Results:**

TCT patients were found to experience AjD in terms of anxiety, depression, and stress, which may have a significant impact on QoL and the recovery/extubation time during long‐term rehabilitation.

**Conclusions:**

The AjD must be considered seriously and carefully during the rehabilitation process in tracheostomized patients, as it can have significant repercussions on the QoL and caregivers. The rehabilitation path must consider the problems linked to this condition: physical, communicative, and psychological.

## Graphical abstract

This graphical abstract visually represents the interconnected relationship between adjustment disorders and overall quality of life, in patients with tracheostomies. The central focus highlights “Quality of Life,” illustrating its significance. Surrounding this core concept are key adjustment disorders, depression, anxiety, and stress, depicted as influential factors. This design emphasizes the critical need to consider these psychological aspects in the comprehensive care and management of tracheostomy patients, ultimately aiming to enhance their overall well‐being and life quality.

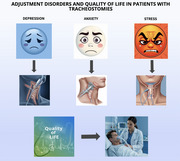

## 1 Introduction

AjD are a group of mental health conditions characterized by emotional or behavioral symptoms that develop in response to a specific stressor or life event, typically occurring within three months of the onset of the stressor. These symptoms are excessive or maladaptive reactions to the stressor, causing significant impairment in social, occupational, or other important areas of functioning (Bachem and Casey 2018; Zelviene and Kazlauskas 2018; O'Donnell et al. 2019; Glaesmer et al. 2015). Their prevalence varies from 5 to 30%, depending on the setting the disorder is diagnosed. Although AjD affects people of all ages, the condition is higher in younger groups, and it affects females more than in males (Carta et al. 2009; Yaseen 2017, Fernández et al. 2012). Diagnosis of AjD is made by means of tools such as structured clinical interviews, including mini international neuropsychiatric interview (MINI), and scales, including the adjustment disorder‐new module 20 (ADNM‐20), which quantify specific reactions to stressors to confirm the diagnosis (Semaan et al. 2001; Liang et al. 2021). Most of the reactions seem to be above the objective severity proportionate to any given stressor; it should be remembered that during life, vulnerability and context modulate the disorder. For example, though vital, life‐saving methods involving respiration through the trachea may be considered a stressor causing AjD, due to the psychological trauma associated with a sudden lifestyle change, dependence, or even anxieties about the condition (Kodicek 1960). Indeed, patients who have undergone a TCT or have a TC can develop AjD. When trying to connect the expressions TCT and TC in AjD patients, it is essential to accurately define both terms: a TCT involves creating an airway by cutting a hole in the neck into the trachea, and the TC is the device placed in this opening to keep the air passage clear. Receiving respiratory assistance through a TC can be extremely troubling for conscious patients who are aware of their reliance on medical devices for essential functions (Engels et al. 2009; Epstein 2005; Mallick and Bodenham 2010; Fernandez et al. 2011). This could result in increased anxiety, signs of depression, or intense stress as individuals try to deal with alterations in independence, daily schedules, and physical appearance, typically seen during extended use of a tracheal tube. Anxiety symptoms can vary from feeling confined with the device to feeling swamped, to even fixating too much on potential issues. Likewise, feelings of reliance, lack of autonomy, and loneliness can lead to depression along with reduced self‐assurance (Howard et al. 2020; Yousefzadeh‐Chabok et al. 2018; Cinotti et al. 2019). Neurologic disorders causing respiratory failure result in changes in consciousness that affect how care is given and received. Coma can result from serious brain injuries such as traumatic brain injury and stroke, rendering patients unresponsive. In these situations, when patients lose consciousness, it becomes challenging to assess their emotional and psychological well‐being since they are unable to express their feelings or acknowledge their condition (Polkey et al. 1999; Neema 2003; Wijdicks and Borel 1998; Ayers 1999; Niedermeyer et al. 2019). In contrast, conditions such as amyotrophic lateral sclerosis or multiple sclerosis can progress with respiratory failure while retaining cognitive function and consciousness; for example, people with amyotrophic lateral sclerosis may be aware of their declining condition but have significantly reduced physical capabilities (Ferreira et al. 2016; Pinto and Carvalho 2014; Calverley 2003). Recognizing this lack of sufficiency may cause deep psychological pain as they try to understand and overcome their limits while being acutely aware of the problem. Similarly, people with severe chronic obstructive pulmonary disease may experience respiratory failure without going unconscious, giving them the opportunity to convey their anxieties and concerns about their situation (MacIntyre and Huang 2008; Gosseries and Laureys 2022). These neurological conditions can also result in coma, as we have said. Right after being revived, comatose survivors may feel confused or disoriented, so they are unable to have an immediate, conscious emotional response to the stressful situation during the rescue. Their psychological responses might only be noticeable as they slowly awaken, making it challenging to differentiate between lingering cognitive issues and actual signs of AjD. This could make diagnosing and treating these patients more difficult since they may appear to be much improved initially, masking the emotional impacts of their illness (Pattison 2005). However, as they develop insight and greater self‐awareness, their psychosocial reactions may become highly intense. On the other hand, patients who stayed awake during the TCT present a completely different set of challenges. The emotional response experienced by these patients is usually complex due to the trauma of tracheal cannulation (Russotto et al. 2022). Being aware that one must depend on a manufactured route for basic respiration induces a strong sense of powerlessness, leading to immediate mental challenges such as fears of inadequate breathing, developing infections, or the device malfunctioning. These worries can become intrusive, causing the individual to constantly check for alarming changes, leading to increased stress and disruptions in sleeping, eating, and communication. In addition to anxiety, a lucid patient may develop profound feelings of depression linked to their TCT (McGiffin et al. 2016; Pandian et al. 2019). An intervention, typically abrupt and lasting, can lead to a profound shift in someone's life and may result in feelings of powerlessness and a loss of sense of self. The patient's physical abilities are restricted, and verbal communication is impaired by the presence of a TCT leading to potential social isolation due to limited social interaction and increased reliance on caregivers (Tippett 2008; Skigen et al. 1999). Depression can occur when individuals feel powerless and experience a decline in self‐esteem as they attempt to adjust to a new reality that differs from their previous expectations and roles in life. This serves as a differentiation factor between an alert patient and a post‐comatose patient in terms of AjD responses in TCT patients. For conscious patients, having a TCT can cause immediate psychological stress that may soon be classified as an AjD. While in individuals who have recently awoken from a coma, the psychological recovery process may be slower and less certain to occur as their cognitive abilities and emotional responses gradually return (Lynch et al. 2020; Gilony et al. 2005; Narayanaswami et al. 2000). Therefore, it will be crucial to employ various techniques, such as providing psychological support to conscious patients in the early stages to handle fear, adapt to changes, and redefine their identity. Having a clear grasp of the mental and emotional hurdles experienced by patients with AjD, especially those going through TCT, is crucial for providing proper care and evaluating their QoL accurately, as it can be greatly affected by physical and emotional aspects. Tools for evaluating health, such as world health organization quality of life brief (WHO‐QOL BREF), offer a wealth of information through various dimensions like physical health, mental well‐being, social interactions, relationships, and overall happiness. QoL surveys indicated that tiredness and difficulty breathing were the primary overall issues, whereas coughing, hearing issues, and speech difficulties were the main symptoms affecting the head and neck region. Patients' answers to the open‐ended questions confirm that the main physical complaints of patients are voice/speech disorders, tracheal secretions, and other respiratory problems (Freeman‐Sanderson et al. 2016; Kumar et al. 2022; Eskildsen et al. 2024).

Although the physical effects of TCT/TC are fairly well‐documented, there remains a notable gap concerning the distinct relationship between AjD and the QoL in these patients, especially in the context of rehabilitation. Current literature frequently discusses psychological distress in broad terms, not specifically isolating or examining the prevalence and effects of AjD. This scoping review is essential for summarizing the existing evidence and pinpointing the particular challenges associated with AjD in TCT/TC patients throughout rehabilitation. Through an analysis of the current research, our goal is to present a detailed summary of the impact of AjD on QoL, thereby emphasizing the necessity for focused psychological interventions. Grasping these dynamics is crucial for creating evidence‐based approaches to enhance the overall welfare and recovery journey of these patients.

This scoping review specifically seeks to investigate how AjD affects the QoL in patients undergoing rehabilitation for TCT or TC. The results will be crucial for creating specific approaches that meet the psychological needs of people with TCT or TC, enhancing their QoL during rehabilitation. Additionally, this review can be valuable for healthcare providers by highlighting the significance of considering both mental and physical aspects of care, enabling holistic treatment strategies that promote patients' well‐being throughout their recovery process.

## 2 Materials and Methods

### 2.1 Search Strategy

This scoping review employed a structured methodology to assess research studies from 2013 to 2023. The investigation encompassed all recent research to gather the current understanding of how AjD influences the QoL in patients with TCT. We performed an extensive literature search through the PubMed, Cochrane Library, and Embase databases, employing the following keywords: (Title/Abstract: “Tracheostomy”) AND/OR (Title/Abstract: “Anxiety”); (Title/Abstract: “Tracheostomy”) AND/OR (Title/Abstract: “Depression”); (Title/Abstract: “Tracheostomy”) AND/OR (Title/Abstract: “Stress”); (Title/Abstract: “Tracheostomy”) AND/OR (Title/Abstract: “Neurorehabilitation”). These databases were meticulously chosen to encompass the widest possible spectrum of peer‐reviewed literature in areas pertinent to this review.

#### 2.1.1 Data Extraction

**FIGURE 1:**
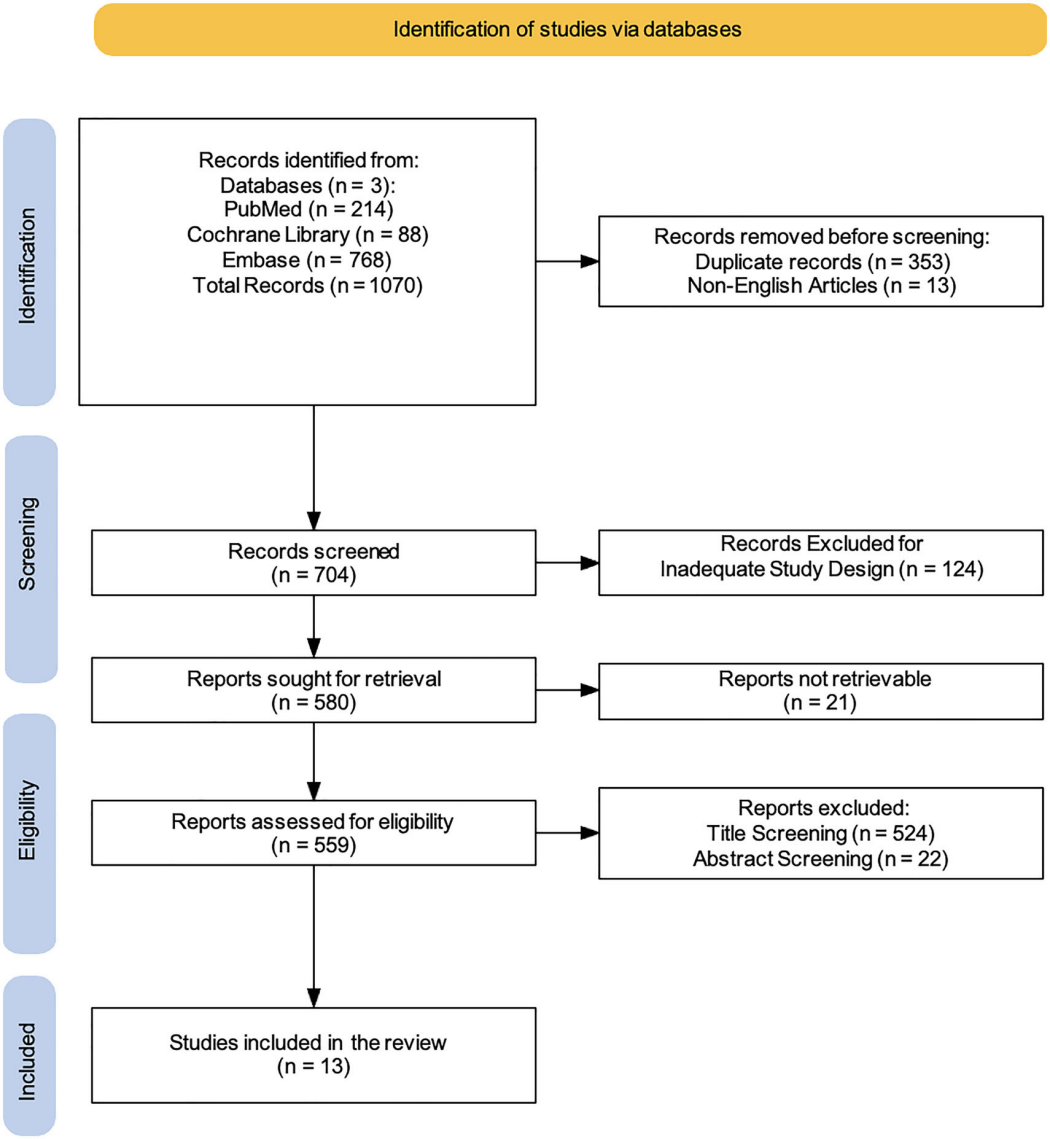
PRISMA 2020 flow diagram of evaluated studies.

Two reviewers (AC, FC) performed independent searches to improve transparency and accuracy in locating pertinent studies. The search strategy was iteratively refined by testing different combinations of keywords, Boolean operators, and controlled vocabulary (e.g., MeSH terms) to maximize sensitivity and specificity. The PRISMA flowchart was employed to depict the process (identification, screening, eligibility, and inclusion) for choosing relevant studies, as shown in Figure 1 (Haddaway et al. 2022). Additionally, two researchers (AC, FC) screened all articles based on titles, abstracts, and full texts, conducting independent data extraction, article gathering, and cross‐validation to minimize bias risks (e.g., missing results bias, publication bias, time lag bias, language bias). The researchers (AC, FC) reviewed complete text articles considered suitable for the study, and if there were disagreements regarding the inclusion and exclusion criteria, a final decision was reached by a third researcher (RSC). Discrepancies between reviewers during the screening or data extraction process were also resolved through discussion, with unresolved cases adjudicated by a third reviewer (RSC). Moreover, the concordance between the two evaluators (AC and FC) was evaluated through the kappa statistic. The kappa score, which has a recognized threshold for significant agreement established at > 0.61, was understood to indicate substantial alignment among the reviewers (McHugh 2012). This standard guarantees a strong assessment of inter‐rater reliability, highlighting the attainment of a significant degree of consensus in the data extraction procedure. The process of data extraction and organization was simplified through the use of microsoft excel, reducing human error and enhancing efficiency. The software facilitated effective handling of extensive datasets, permitting reviewers to methodically document study attributes, evaluations of bias risk, and outcome information. Custom extraction sheets were created in the software to guarantee uniformity and compliance with the established inclusion/exclusion criteria. Moreover, the software offered functionalities like tagging, filtering, and sorting, which helped in resolving discrepancies and speeding up the cross‐validation procedure. The collection of articles was later improved for relevance, evaluated, and summarized, with key topics emphasized from the summary based on the inclusion/exclusion criteria. This scoping review has been registered on Open OSF with the following DOI number: DOI 10.17605/OSF.IO/RHX3J.

#### 2.1.2 Data Synthesis

Data synthesis was conducted using narrative methods to tackle the variety of study designs and types of conditions of TCT patients. This method allowed us to identify key themes, commonalities, and differences across the research landscape. By synthesizing qualitative observations, we provided a comprehensive understanding of the evidence related to AjD and TCT. During the synthesis process, the involvement of a multidisciplinary team guaranteed an even interpretation of the data. Frequent discussions and consensus meetings among reviewers aided in reducing potential biases in qualitative evaluations and ensured uniformity in categorizing and interpreting results.

### 2.2 PICO Evaluation

We defined our combination of search terms using a PICO (population, intervention, comparison, and outcome) model. The population of this scoping review varies from those in critical care to individuals with chronic conditions. Interventions encompass the TCT procedure itself, along with various care strategies like communication aids, specialized nursing, and educational programs. Comparisons were made between TCT and non‐TCT groups, standard versus enhanced care, and pre‐ versus post‐intervention scenarios. Primary outcomes measured include QoL, functional abilities, and psychological well‐being, with a focus on AjD.

### 2.3 Inclusion Criteria

This scoping review encompassed research that investigated the impact of TCT among various patient groups. In particular, research that included people who received TCT for various medical reasons, such as malignant lesions, extended mechanical ventilation, head and neck cancer, neuromuscular disorders, acute respiratory distress syndrome, and serious traumatic brain injuries, was taken into account. Studies that met the criteria evaluated outcomes concerning the patient's QoL, AjD, functional ability, and psychological wellness. This encompassed assessments of anxiety, depression, post‐traumatic stress, and overall emotional adaptation. Research examining the effects of interventions intended to enhance TCT care, including communication tools, specific nursing guidelines, and training programs, was also incorporated. Both observational and interventional study designs were taken into account, comprising randomized controlled trials, prospective and retrospective cohort studies, cross‐sectional analyses, and qualitative research. Research reported on results assessed using verified scales or well‐defined metrics. To maintain clarity and consistency, only articles published in English that are full‐text were included. Research distinctly outlined its methodologies, the demographics of participants, and the precise elements of TCT care or patient experience that were assessed. Ultimately, research explored the relationship between TCT and psychological adaptation, highlighting its effect on the patient's overall QoL.

### 2.4 Exclusion Criteria

Studies were excluded if they did not specifically focus on the impact of TCT on patient outcomes, particularly concerning QoL, functional status, and psychological well‐being. Research investigating only the technical aspects of TCT procedures or solely focusing on physiological parameters, without addressing patient‐reported outcomes or psychological factors, was omitted. Additionally, studies that did not address relevant outcomes related to psychological adjustment, such as anxiety, depression, or post‐traumatic stress, were excluded. Studies that failed to define participant groups clearly, or those that included a mix of patients with various conditions without specifically isolating those who underwent TCT, were also excluded to maintain consistency in the analysis. Furthermore, study protocols, conference abstracts, editorials, and reviews, whether systematic, narrative, or integrative, were not included. Finally, research published in languages other than English or those without full‐text access was excluded to ensure a thorough and methodologically rigorous evaluation.

## 3 Results

A comprehensive literature search was carried out using three electronic databases. This initial search yielded a total of 1070 records. Before formal screening commenced, 353 duplicate records were identified and removed, along with 13 non‐English articles. This left 704 records for title and abstract screening. Following this initial screening phase, 124 records were excluded based on inadequate study design, leaving 580 reports for full‐text retrieval. However, despite efforts to obtain these articles (e.g., emailing corresponding authors, consulting libraries, exploring open‐access sources, checking institutional resources, and using research networks), 21 reports could not be retrieved. The remaining 559 reports underwent a thorough assessment for eligibility. During this stage, 524 reports were further excluded based on title screening, and an additional 22 were excluded after abstract screening. This rigorous selection process resulted in a final set of 13 studies that met the pre‐defined inclusion criteria (Phookan and Talukdar 2023; Haring et al. 2022; Breckenridge et al. 2014; Volpato et al. 2023; Jacobs et al. 2021; Blecha et al. 2020; Rasool et al. 2023; McGrath et al. 2020; Yin et al. 2021; Mehta et al. 2022; Bozec et al. 2021; Freeman‐Sanderson et al. 2016), and were therefore included in this review (table 1).

### 3.1 Demographic and Aetiological Characteristics: Race, Sex, and Contributing Factors in TCT Populations and Their Geographic Distribution

Research carried out in different geographic regions showed diverse demographic and etiological traits among TCT populations. A single‐center study in India indicated that malignant lesions were the main cause, representing 82.47% of cases, with most patients receiving emergency temporary TCTs (Phookan and Talukdar 2023). In Canada, a multicenter investigation with 534 patients (391 in the one‐year group) revealed that TCT patients, among whom 193 lived for one year, underwent extended mechanical ventilation and hospital durations, indicating TCT as an indicator of illness severity (Bozec et al. 2021). In Minnesota, USA, a research study with 116 mechanically ventilated patients (51 undergoing TCT) revealed no notable differences in age, gender, race, or ethnicity when comparing TCT and non‐TCT groups (Breckenridge et al. 2014). A study involving 22 patients with neuromuscular diseases in Italy revealed psychological distress amid the COVID‐19 pandemic (Volpato et al. 2023). In Jerusalem, Israel, an observational study involving 62 patients receiving prolonged mechanical ventilation found that 43.5% had neuromuscular disorders and 29% had chronic lung diseases (Jacobs et al. 2021). A German cohort study involving 388 survivors of ARDS indicated that TCT had no notable effect on long‐term QoL (Blecha et al. 2020). A study conducted in Lahore, Pakistan, involving 34 patients who had permanent TCTs found that 70.6% of them were male, and 44% were in the 41–50 age range (Rasool et al. 2023). In the UK, research with 2405 patients indicated notable decreases in both ICU and hospital durations (McGrath et al. 2020). In China, a study was conducted on 60 patients with severe traumatic brain injuries (Yin et al. 2021). In France, research involving 48 laryngectomy patients indicated that elevated education levels were associated with poorer voice handicap index results (Bozec et al. 2021).

### 3.2 Study Design, Research Methods, and Data Collection Tools

Across the studies reviewed, a variety of research designs and data collection tools were employed to investigate the impact of TCT. Observational studies, such as Phookan et al. (Phookan and Talukdar 2023), utilized the UWQOL v4 to assess QoL in 97 patients, revealing persistent challenges with anxiety and voice production post‐TCT. Uncontrolled experimental designs were also prevalent; for example, (Haring et al. 2022), evaluated the efficacy of an iPad communication app in 35 head and neck cancer patients using researcher‐developed surveys and the V‐RQOL, demonstrating a significant reduction in communication difficulties. Similarly, (Breckenridge et al. 2014), conducted an uncontrolled experimental study on 166 mechanically ventilated patients, using the VAS‐A to find that TCT did not significantly reduce anxiety. Qualitative research, exemplified by (Volpato et al. 2023), explored the experiences of 22 neuromuscular disease patients through interviews and psychological tests, highlighting feelings of isolation and distress. Observational studies like (Jacobs et al. 2021), examined 62 patients with prolonged mechanical ventilation using r‐ESAS and GDS, finding a high prevalence of depression. Prospective cohort studies, such as (Blecha et al. 2020), followed 1205 ARDS patients using HRQoL and psychiatric symptom measures, reporting no significant impact of TCT on these outcomes. Interventions, such as the educational program in (Rasool et al. 2023), involving 34 patients, used self‐care questionnaires and HADS, showing improved self‐management and reduced anxiety. (McGrath et al. 2020), implemented a quality improvement program in 20 UK hospitals, assessing 2405 patients through patient safety incidents, HADS, and NoMAD, revealing significant improvements in patient outcomes. (Yin et al. 2021), in China compared traditional nursing care with HCH nursing care in 60 TBI patients using GCS, Karnofsky scores, and SAS, finding improved functional status and reduced anxiety in the HCH group. Mehta et al. (2022), conducted a prospective cohort study on 534 patients in Canada, using FIM and PFTs, finding increased care burden and worse functional outcomes in TCT patients. (Bozec et al. 2021), in France conducted a cross‐sectional study on 48 laryngectomy patients using EORTC and HADS, revealing psychological distress as a key predictor of poor outcomes. Finally, Sanderson et al. (Freeman‐Sanderson et al. 2016), in Australia conducted both observational and randomized controlled trials, using VASES and EQ‐5D to assess the impact of voice restoration and early speech‐language pathology intervention on quality of life.

#### 3.2.1 Safety and Adverse Events

Adverse events were indicated by prolonged care and functional decline across several studies. In Canada, TCT patients experienced significantly longer mechanical ventilation, ICU, and hospital stays, with a higher hospital mortality rate (OR 2.6, *p* = 0.03) (Mehta et al. 2022). Functional Independence Measure (FIM) scores were significantly lower, indicating functional decline post‐TCT (FIM Motor Subscale *p* = 0.0002; FIM Total *p* = 0.0004). ICU readmission was also more frequent (*p* = 0.04). In India, anxiety remained a persistent issue post‐TCT (Phookan and Talukdar 2023). In Minnesota, USA, sedative medication use increased significantly post‐TCT (*p* < 0.001) (Breckenridge et al. 2014). In Germany, TCT was associated with longer ICU stays (*p* < 0.001) (Blecha et al. 2020). In Israel, 38% of patients screened positive for depression (GDS ≥ 2/5) (Jacobs et al. 2021). In Italy, neuromuscular disease patients reported heightened psychological distress (Volpato et al. 2023). In China, patients receiving traditional care experienced higher incidences of artificial TC blockage and readmission than those receiving HCH care (*p* < 0.05) (Yin et al. 2021). In the UK, patient safety incident severity decreased after the implementation of a structured quality improvement program (*p* < 0.001) (McGrath et al. 2020).

### 3.3 Impact of Anxiety, Depression and Stress on QoL in TCT Patients

In various studies, the psychological effects of TCT on QoL consistently stand out as a major issue, with anxiety, depression, and stress all having considerable influences. Significantly, anxiety frequently remains after TCT, as indicated in Indian research displaying minimal progress (Phookan and Talukdar 2023), and in Minnesota, where levels varied each day (Breckenridge et al. 2014). Communication challenges, especially in Michigan, were a significant cause of stress and frustration (Haring et al. 2022), whereas in Italy, patients with neuromuscular diseases faced increased psychological distress, including anxiety, during the pandemic (Volpato et al. 2023). Depression also impacts TCT patients, as a significant percentage exhibit positive screening results in Israeli research (Jacobs et al. 2021), and psychological distress, which includes depressive symptoms, is a major predictor of adverse outcomes in French laryngectomy patients (Bozec et al. 2021). While a German research indicated no notable effect on psychiatric symptoms, such as depression (Blecha et al. 2020), the abundant evidence highlights depression's considerable impact on quality of life. Stress, expressed through extended caregiving, a decrease in functioning, and feelings of emotional solitude, adds to these difficulties. Canadian research emphasizes the stress linked to prolonged hospitalizations and diminished functional independence (Mehta et al. 2022). The issues of emotional isolation and continuity of care noted in Italy highlight the widespread stress experienced by this patient group (Volpato et al. 2023). Optimistically, organized interventions, like those carried out in the UK and Pakistan, show promise in alleviating these psychological challenges, lowering the rates of both anxiety and depression (Rasool et al. 2023, McGrath et al. 2020), indicating that focused support can substantially enhance the overall well‐being of patients with TCTs.

### 3.4 Mitigation Strategies in Patients With TCT and QoL

Several mitigation strategies demonstrated positive impacts on QoL in TCT patients. In India, targeted interventions were recommended to address employment, anxiety, and voice production, which showed limited improvement post‐TCT (Phookan and Talukdar 2023). In Michigan, electronic communication devices significantly reduced self‐reported communication difficulties from 89% to 53% (*p* = 0.03) and improved treatment/discharge plan communication from 78% to 35% (*p* < 0.01) (Haring et al. 2022). In Pakistan, an educational intervention significantly improved self‐care knowledge and reduced anxiety levels (*p* < 0.000) (Rasool et al. 2023). In the UK, a structured quality improvement program reduced anxiety prevalence by 44.3% (*p* = 0.008) and depression by 52.7% (*p* < 0.001), while also reducing ICU and hospital stays (McGrath et al. 2020). In China, Hospital‐Community‐Home (HCH) nursing care led to significantly higher functional status scores and lower caregiver anxiety (*p* < 0.05, *p* = 0.009) (Yin et al. 2021). Voice restoration in Australia improved “being understood” (*p* = 0.006) and “cheerfulness” (*p* = 0.042) (Freeman‐Sanderson et al. 2016). Early speech‐language pathology intervention in Australia significantly reduced time to phonation (*p* = 0.001) (Freeman‐Sanderson et al. 2016). Finally, in Israel, home‐based care was associated with better well‐being and less depression (*p* = 0.049) (Jacobs et al. 2021).

## 4 Discussion

A qualitative analysis of the reviewed studies underscores the pervasive impact of AjD on the QoL of TCT patients, yet reveals a complex interplay of variables. Phookan et al.’s Indian study, with its high malignancy rate (82.47%), suggests anxiety and voice production remain stubbornly resistant to improvement, even as overall QoL improves (Phookan and Talukdar 2023). Mehta et al., in their study, with large cohorts, highlight the selection bias inherent in TCT; it's often a marker of severe illness, confounding interpretations of prolonged care and functional decline (Mehta et al. 2022). Haring et al., show that communication interventions can dramatically reduce anxiety (55% to 53%, *p* = 0.03) and depression (44% to an unstated lower percentage), but the small sample (*n* = 35) limits generalizability (Haring et al. 2022). Breckenridge et al., deliver a crucial counter‐narrative: anxiety doesn't inherently decrease post‐TCT, and sedative use increases, emphasizing the need for ongoing psychological support (Breckenridge et al. 2014). Volpato et al., highlight the psychological vulnerability of neuromuscular disease patients during the pandemic, with rich qualitative data, yet limited quantitative reach (Volpato et al. 2023). Jacobs et al., suggest that home‐based care improves well‐being, but the focus on communicative patients skews the findings (Jacobs et al. 2021). Blecha et al., stand out, showing no long‐term impact on psychiatric symptoms (*p* > 0.05), challenging the prevailing narrative, but it's focused on ARDS survivors (Blecha et al. 2020). Rasool et al. and McGrath et al., demonstrate the effectiveness of targeted interventions in reducing anxiety and depression, respectively (Rasool et al. 2023; McGrath et al. 2020). Yin et al., show HCH care improves functional status and reduces caregiver anxiety (Yin et al. 2021). Bozec et al. show psychological distress (HADS ≥ 15) is the main predictor of poor QoL post laryngectomy (Bozec et al. 2021). Sanderson et al. (2016) Australian studies show SLP intervention improves QoL and time to phonation (Freeman‐Sanderson et al. 2016). Ultimately, these studies reveal that while TCT often improves physiological function, the associated psychological burden requires targeted, context‐specific interventions to truly enhance QoL.

### 4.1 Persistent Challenges in Tracheostomy Recovery: Communication, Reintegration and QoL

Analyzing these research studies (Phookan and Talukdar 2023; Haring et al. 2022; Breckenridge et al. 2014; Volpato et al. 2023; Jacobs et al. 2021; Blecha et al. 2020; Rasool et al. 2023; McGrath et al. 2020; Yin et al. 2021; Mehta et al. 2022; Bozec et al. 2021; Freeman‐Sanderson et al. 2016; Freeman‐Sanderson et al. 2016) on TCT reveals a coherent story about the diverse effects on patient health. It is clear that the process, although frequently essential from a medical standpoint, triggers a series of physiological and psychosocial changes (Rovira et al. 2021; Lais and Piquilloud 2024). The variety of causes, from cancerous tumors to extended use of mechanical ventilation, highlights the different patient demographics seen in clinical settings (Bonvento et al. 2017; Sherlock et al. 2009; Marchese et al. 2010). A common motif across these studies is the ongoing presence of specific challenges, particularly anxiety, voice production, and vocational reintegration, even amid general enhancements in quality of life. This observation raises an important question about the effectiveness of existing rehabilitation procedures. Are we sufficiently tackling these particular areas, or are there shortcomings in our treatment approaches? The occurrence of anxiety, as reported in various studies, requires special consideration (Sutthipongkiat et al. 2024; Liney et al. 2019; Fuller et al. 2021). The noted variations in anxiety ratings and the corresponding rise in sedative medication use indicate a substantial psychological strain (Reade and Finfer 2014). This requires a more subtle method for managing anxiety, possibly integrating evidence‐supported psychological therapies with pharmacological treatments. Moreover, the effect of communication difficulties cannot be underestimated. The use of electronic communication devices, as shown in the Michigan study, signifies a hopeful path for alleviating these issues (Haring et al. 2022). Nonetheless, it is essential to reflect on the wider effects of communication challenges on patient independence and social inclusion (McClintock et al. 2024; Tolotti et al. 2023,;Newman et al. 2022). The caregiver burden highlights the systemic characteristics of TCT care (Luo et al. 2025). Successful interventions should reach beyond the patient to include the support system (Aung et al. 2025). This requires a comprehensive strategy that considers the requirements of both patients and their caregivers (Nakarada‐Kordic et al. 2017). It is equally important to recognize the differences in research results. For example, the German research showing no lasting effects on psychiatric symptoms offers a differing viewpoint (Blecha et al. 2020). This emphasizes the significance of framing research results and taking into account the unique traits of the study group. In conclusion, these studies together highlight the intricate nature of TCT care. A thorough, patient‐focused strategy is crucial, incorporating physiological, psychological, and social factors. This requires a cooperative, cross‐disciplinary approach that emphasizes personalized treatment and continual assessment of treatment effectiveness. Additionally, it is essential to enhance our comprehension of the prolonged effects of TCT and create focused strategies to improve patient results.

### 4.2 Future Directions in Tracheostomy Care: Tailoring Rehabilitation and Psychosocial Support

The gathered studies on TCT patients present a story of medical progress alongside ongoing difficulties. Although TCT frequently offers essential physiological assistance, it also brings about a complicated set of psychosocial factors that require our focus (Pierucci et al. 2023). In the future, various important areas arise as essential to research and clinical progress. Above all, the necessity for individualized rehabilitation cannot be emphasized enough. The differences in patient experiences, from the root cause of the TCT to personal psychological strength, underscore the shortcomings of a universal approach. Future initiatives should concentrate on creating customized rehabilitation strategies that integrate patient‐reported results in real time. This will enable more responsive and efficient interventions. Moreover, ongoing psychosocial assistance is crucial. The continued presence of anxiety and depression in numerous studies highlights the necessity for enduring support systems that follow hospital discharge (Pinho et al. 2021). Cutting‐edge care approaches, including telehealth and peer support systems, show potential for closing this gap (Bulkes et al. 2022). Communication technologies additionally present considerable potential. Although electronic devices have proven to be effective, achieving fair access and providing training continue to pose a challenge (Brenner and Pandian 2024). Future studies should investigate how technology can be utilized to improve social engagement and diminish feelings of isolation. Furthermore, the significance of caregiver assistance must not be ignored. Caregivers hold an essential position in the healing process of patients, and it's important to meet their psychological and practical needs (Şapulu Alakan et al. 2023). Regarding research methodologies, upcoming studies should focus on reducing confounding variables and employing a mix of quantitative and qualitative methods. Standardized outcome measures will be crucial for comparing findings across different studies. Finally, enhancing emergency TCT techniques is a research area that has the potential to significantly benefit patient results. Investigating the efficiency, safety, and education of healthcare personnel could assist in optimizing and enhancing the effectiveness of emergency TCTs. By focusing on these crucial aspects, future studies and clinical practices can advance toward a more comprehensive and patient‐focused method of TCT care, ultimately enhancing the sustained QoL for those undergoing this procedure.

### 4.3 Strenghts and Limitations

This scoping review utilizes a rigorous method to synthesize literature regarding the effects of TCT on QoL, especially concerning AjD, revealing significant strengths as well as inherent weaknesses. The review's advantages stem from its organized and thorough search approach, covering various pertinent databases and utilizing a clearly defined search string. The thorough two‐step screening method, along with a third reviewer resolving disagreements and the application of the PRISMA flowchart, improve the dependability and clarity of study selection. Nonetheless, the variability among the studies included, featuring patient groups that varied from those with malignant lesions to those with neuromuscular disorders, restricted the opportunities for meta‐analysis. Moreover, biases in language and publication, alongside differences in outcome measures, created obstacles for directly comparing the findings. Although a focus on geographic diversity was intended, an unequal number of studies came from Canada and the UK, and racial and ethnic information was limited, with the Minnesota study serving as the main reference, showing no notable differences in these categories between TCT and non‐TCT groups. The lack of control groups in multiple studies and the restricted access to long‐term data also limited the breadth of our conclusions. Factors like pre‐existing health issues and the inability to access records might have affected the results observed. Ultimately, the emphasis on AjD, though offering significant insight, limited the investigation into other possible psychological effects. The limited number of studies that satisfy the inclusion criteria, even after an exhaustive search, greatly restrict the generalizability of the results and hinders the capacity to make conclusive determinations. The lack of data highlights the essential requirement for more research in this overlooked field. Consequently, although this review provides a useful synthesis, additional research utilizing more uniform methodologies and varied populations is necessary.

## 5 Conclusion

In conclusion, this analysis demonstrates the significant influence of AjD on the QoL of patients undergoing rehabilitation after TCT. The emotional and mental health of patients becomes important aspects of their recovery as numerous individuals encounter communication obstacles that result in feelings of frustration and anxiety. Research shows that using efficient communication tools can significantly reduce these emotional stressors and improve QoL. Nevertheless, even with these improvements, a significant number of patients still struggle with anxiety and depression, mainly because surgeries are rushed and there is not enough preoperative counseling. These difficulties underscore the need for holistic care that includes emotional assistance during the healing journey. Future research needs to focus on tailored rehabilitation approaches that consider individual age and functional capabilities while also acknowledging the specific emotional challenges patients face. Healthcare providers can improve patient outcomes and overall well‐being by considering both psychological and physical aspects of recovery through holistic approaches. In the end, creating a supportive environment that puts the emotional well‐being of patients first is crucial for enhancing rehabilitation outcomes and enhancing the overall QoL for TCT patients Table 1.

**TABLE 1:** Summary of the included studies.

Author/location/ country of the study	Aim	Study design/sample size/demographic data/diagnosis	Treatment period	Intervention/control group	Outcomes measures	Main findings	Adjustment disorder/connection adjustment disorder and Quality of life
Phookan et al. 2023 (Phookan and Talukdar 2023) Location: hospital. Country: India.	To assess the QoL in individuals who had a TCT performed.	Study Design: Observational Study. Size: 97 patients. Age: from under 20 to over 60 years. Sex: Male: 81 patients (83.51%); Female: 16 patients (16.49%). Diagnosis: the primary diagnosis necessitating TCT was malignant lesions (82.47%).	The research monitored patients for 12 weeks after their tracheostomy. The overall length of the research was one year.	As this was an observational study, there were no separate IG or CG.	UWQOL v4.	Malignant lesions, particularly in the supraglottic area, were the primary cause of tracheostomies, which were primarily urgent procedures and short‐term measures. Although QoL enhanced over 12 weeks, patients continued to encounter difficulties with work, anxiety, and voice production, especially during the initial recovery phase. The COVID‐19 pandemic negatively impacted the QoL for patients with head and neck cancer.	Adj: anxiety, mood distubances, and social isolation. Connection Adj and QoL: experiencing a TCT is a significant life event that can cause emotional turmoil because of alterations in physical ability, appearance, and daily care requirements. Numerous patients faced psychological issues, such as anxiety, mood changes, social withdrawal, and struggles with employment and everyday tasks. Although physical recovery improved, emotional, and social challenges continued, emphasizing the necessity for ongoing support.
Haring et al. 2022 (Haring et al. 2022) Location: university of Michigan department of otolaryngology–head and neck surgery. Country: United States.	To assess the effect of utilizing an electronic communication application (Verbally on an iPad) on the communication experience and quality of life of patients who have had a TCT or total laryngectomy for HNC. In particular, it aimed to evaluate whether this intervention could alleviate communication challenges and lessen related discomfort.	Study Design: Uncontrolled Experimental Study. Size: 35 patients, divided into two groups: 18 patients in the pre‐intervention group and 17 patients in the post‐intervention group. Age: Pre‐intervention: average age of 64 years; Post‐intervention: average age of 63 years. Sex: Pre‐intervention: 83% male (15/18); Post‐intervention: 88% male (15/17). Diagnosis: patients participating in the study had either a TCT or total laryngectomy performed for the management of HNC.	The intervention phase concentrated on the early postoperative period, with surveys given on postoperative days three to five. The intervention, which involved providing the iPad, commenced on the first postoperative day. Data was gathered from april 2018 to december 2019.	IG: Pre‐intervention group: 18 patients (using whiteboard and pen). Post‐intervention group: 17 patients (using iPad with communication application). CG: not present.	A researcher‐developed survey assessing patient‐reported communication difficulties and V‐RQOL.	Patients in the post‐intervention group indicated a notable reduction in communication challenges, especially about treatment and discharge plans. Challenges reported by participants decreased from 89% to 53%, underscoring the intervention's success. Without the iPad, numerous patients faced frustration, anxiety, or depression because of communication obstacles.	Adjustment Disorder: anxiety and depression related to communication difficulties. Connection Adj and QoL: the research emphasizes the connection between communication challenges following TCT or laryngectomy and heightened emotional distress, affecting patients' QoL. It shows that employing an iPad with a communication app greatly lessens frustration, anxiety, and depression by enhancing communication. These results indicate that electronic devices may significantly assist in promoting mental well‐being throughout the recovery process.
Breckenridge et al. 2014 (Breckenridge et al. 2014) Location: Minneapolis and St. Paul, Minnesota urban area. Country: United States.	To assess whether the placement of TCT lessens anxiety levels in critically ill patients on MV. It involved a secondary analysis of data derived from a parent study that explored the impact of music listening on anxiety.	Study Design: Uncontrolled Experimental Study. Size: 166 patients. Age: 18 years or older. Sex: not specificated. Diagnosis: Patients were MV in the ICU, primarily for respiratory or cardiac issues.	Participants stayed on the protocol while receiving MV in the ICU, ranging from 1 to 30 days as per protocol.	IG: 51 patients underwent TCT placement. CG: 65 patients did not undergo TCT placement.	VAS‐A. Sedation was measured by recording the receipt of sedative medications. Time to TCT was also recorded.	The placement of TCT did not notably lessen anxiety in critically ill patients, and the administration of sedative drugs rose subsequently. Age was the sole factor affecting anxiety levels over time, whereas daily changes in anxiety were significant.	AjD: anxiety. Connection Adj and QoL: While the research centered on anxiety, it indicates that the QoL might not enhance as anticipated following TCT. The absence of decreased anxiety and heightened sedation suggests that patients might continue to undergo considerable distress and discomfort.
Volpato et al. 2023 (Volpato et al. 2023) Location: centers in Milan and Bari. Country: Italy.	To examine the experiences of individuals with NMD and CRF who were using TCT and HIMV amid the COVID‐19 pandemic.	Study Design: Qualitative Study. Size: 22 participants. Age: mean age of the participants was 50.2 years. Sex: Men: 9 (40.9%) Women: 11 (50%) Diagnosis: ALS, SMA, DMD, congenital, myopathies, miasthenia, gravis And muscular distrophy.	Data were collected from december 10, 2021 to april 30, 2022.	Not applicable as this was a qualitative study.	Demographic and clinical data were collected. CD‐RISC‐25. AAQ‐II, STAI, LMS.	Increased dispositional mindfulness correlated with enhanced resilience, yet numerous patients faced feelings of abandonment, isolation, and insufficient support. Decreased interaction with healthcare professionals and insufficient continuity of care decreased satisfaction, while TCT was perceived as both life‐preserving and anxiety‐inducing. Furthermore, the insufficient use of open ventilation, phonation valves, and cough machines indicates potential chances to enhance QoL.	AjD: anxiety, feelings of abandonment, isolation, and distress. Connection Adj and QoL: The research showed notable declines in QoL attributed to isolation, less healthcare interaction, and difficulties in communication. The physical strain of TC and HIMV, combined with the restricted availability of supportive tools such as phonation valves and cough machines, negatively impacted patient well‐being. These issues were heightened by the pandemic, intensifying the struggles experienced by those with NMD and CRF.
Jacobs et al. 2021 (Jacobs et al. 2021) Location: Jerusalem Country: Israel	To characterize the emotional state, symptoms, health status, and perceptions of ventilation among patients receiving PMV through TCT, whether at home or in LTAC facilities.	Study design: observational study Size: 62 participants. Age: mean age of the participants was 61.7 years Sex: Male: 34 (54.8%); Female: 28 (45.2%) Diagnosis: progressive degenerative neuromuscular disease (43.5%); chronic lung disease (29%); neurologic acute (16.1%); post sepsis (14.5%). heart disease (4.8%).	Between may 1, 2016, and april 31, 2018.	There were no IG or CG. The study compared patients receiving PMV at home (40 participants) versus those in LTAC (22 participants).	Sociodemographic characteristics; Primary indication for PMV. Current comorbidities and medications; functional status (Barthel Index); daily hours and duration of ventilation; r‐ESAS; GDS. Attitudes toward ventilation.	The majority of participants exhibited significant dependence and needed ventilation for more than 18 h each day, but the overall symptom burden remained low despite regular impairments in well‐being. Depression impacted 38% of patients, with improved mental well‐being noted in those treated at home. Though few possessed advance directives, the majority would opt for PMV once more, although chronic lung disease was associated with increased symptom severity.	AjD: anxiety and depression. Connection Adj and QoL: The research underscores a significant connection among psychological adjustment, QoL, and symptom burden, indicating that depression and anxiety greatly affect well‐being. The care environment was vital, indicating that surrounding conditions affect adjustment and psychological well‐being. Even with serious medical issues, numerous patients expressed satisfaction with life, highlighting the significance of psychological assistance in enhancing quality of life for individuals on PMV.
Blecha et al. 2020 (Blecha et al. 2020) Location: multiple ICUs Country: Germany.	To investigate the effect of TCT on HRQoL, the emergence of psychiatric symptoms, and return‐to‐work in survivors of acute respiratory distress syndrome (ARDS) one year after being discharged from the ICU.	Study Design: Prospective Observational Study. Size: 1205 ARDS patients for initial analysis. Age: mean age of the participants was 56 years (±15.8). Sex: Men: 68%; Women: 32%. Diagnosis: participants were diagnosed with ARDS according to the Berlin definition.	The research involved patients who received treatment from September 2014 to April 2016. Follow‐up surveys were conducted at 3, 6, and 12 months following ICU discharge.	IG: TCT group: 659 patients. CG: Non‐TCT group: 546 patients.	HRQoL; SF‐12; PCS‐12; MCS‐12; PHQ‐9; PTSS‐14; Return‐to‐work status.	TCT showed no notable effect on PCS‐12 or MCS‐12 scores, psychiatric symptoms, or work resumption rates after a year. Nonetheless, the duration of ICU stays was significantly greater for patients who underwent TCT.	AjD: Depression and anxiety. Connection Adj and QoL: The research investigated the influence of tracheostomy on psychiatric symptoms and HRQoL among ARDS survivors, revealing no notable effect on PTSD, depression, or general well‐being. Although patients with tracheostomies had extended stays in the ICU, their QoL was similar to that of those without TCT. These results emphasize the intricate connection between psychological adaptation and physical well‐being during recovery.
Rasool et al. 2023 (Rasool et al. 2023) Location: general hospital, lahore. Country: Pakistan.	To evaluate the impact of a TCT care program on self‐management and anxiety levels in adult patients with long‐term TCT.	Study design: uncontrolled experimental Study. Size: 34 participants. Age: 18–30 years: 10 (29.4%); 31–40 years: 9 (26.5%); 41–50 years: 15 (44%). Sex: Male: 24 (70.6%); Female: 10 (29.4%) Diagnosis: Participants had permanent TCT.	The research was carried out following the approval of the institutional review board (IRB) at the lahore university of lahore, beginning on january 25, 2022.	IG: 34 patients. CG: not present.	Self‐care questionnaire with 16 yes/no questions and HADS.	The educational intervention greatly improved self‐care understanding and behaviors in individuals with permanent TCT. It also resulted in a significant decrease in anxiety, with elevated pre‐intervention anxiety levels dropping considerably afterward.	AjD: anxiety. Connection Adj and QoL: The research demonstrated that increasing self‐care knowledge via education notably lowered anxiety levels in individuals with permanent TCT. This indicates that equipping patients with the abilities to handle their condition enhances psychological adaptation and general wellness.
McGrath et al. 2020 (McGrath et al. 2020) Location: England, Wales, and Scotland. Country: United Kingdom.	To enhance the quality and safety of TCT care by systematically implementing interventions across various NHS hospitals in the United Kingdom.	Study Design: Observational study. Size: 2405 discrete patient admissions. Age: average age of the participants was 50.1 years. Sex: Male: 60.2%; Female: 34.6%; N/A: 5.2% Diagnosis: Patients requiring TCT care.	Hospital admissions were recorded from august 1, 2016 (month 0) to january 31, 2018 (month 30).	This was an observational study involving 20 participating sites, not a traditional IG/CG setup. All 20 sites participated in the quality improvement program.	Patient safety incidents and severity. Length of stay (hospital, ICU). Ventilator days. TCT days. Time to first vocalization and oral intake. HADS; NoMAD. Economic evaluation of cost of care.	The research showed notable enhancements in patient results, such as decreased ICU and hospital durations, quicker restoration of speech and eating, and diminished anxiety and depression levels. Moreover, occurrences of patient safety incidents declined, and staff engagement increased, resulting in significant cost reductions.	AjD: anxiety and depression. Connection Adj and QoL: The research indicated that improved tracheostomy care, which involved shorter hospital visits and better communication, notably lowered levels of anxiety and depression. This emphasizes the significant connection between clinical care and psychological health in patients with TCT. Quicker recovery of speech and oral intake also led to an improved QoL. In general, enhancing care directly benefited both mental well‐being and overall patient results.
Yin et al. 2021 (Yin et al. 2021) Location: Suzhou science and technology town hospital of Nanjing medical university. Country: China.	To assess the efficacy of HCH nursing care in relation to traditional nursing care for TCT patients experiencing severe TBI.	Study Design: Uncontrolled experimental study. Size: 60 patients, with 30 patients in the traditional nursing care group and 30 patients in the HCH nursing care group. Age: not specificated. Sex: not specificated. Diagnosis: Patients were diagnosed with severe TBI and required TCT.	Patients were enrolled from january 2018 to december 2019. Patients enrolled from january 2018 to december 2018 were provided with conventional nursing care. Individuals enrolled between january 2019 and december 2019 were provided with HCH nursing care. A follow‐up was performed two months after discharge.	IG: 30 patients. CG: 30 patients.	GCS, Karnofsky Performance Status Scale, SAS, Barthel Index; TCT‐related complications. Readmission rates.	At the two‐month follow‐up, the HCH nursing care group demonstrated notably improved results, featuring higher GCS, Karnofsky scores, and Barthel Index scores in comparison to the traditional care group. Caregivers in the HCH group indicated notably reduced anxiety levels. Moreover, the HCH group experienced a lower number of tracheal cannula obstructions and readmissions. The groups did not show significant differences regarding subcutaneous emphysema, pulmonary infections, or incision infections.	Adjustment Disorder: anxiety. Connection Adj and QoL: The research indicated that HCH nursing care led to better functional status (Karnofsky, Barthel) and neurological recovery (GCS) for patients, thereby directly improving their QoL. Moreover, the decrease in caregiver anxiety (SAS) indicates that the HCH model also promotes the mental health of caregivers, enhancing the entire support network. By reducing complications and readmission rates, the HCH care plan enhances the QoL for both patients and caregivers.
Metha et al. 2022 (Mehta et al. 2022) Location: nine university‐affiliated ICUs in Toronto, Hamilton, Ottawa, Montreal, Sherbrooke, and Vancouver. Country: Canada.	To assess the results of TCT in individuals who underwent a minimum of one week of MV and were monitored for one year.	Study design: prospective cohort study. Size: 534 patients, with 391 included in the one‐year follow‐up cohort. Age: Patients were 16 years old or older. Sex: not specificated. Diagnosis: patients were dependent on MV in the ICU for 7 days or more.	The research took place from february 2007 to march 2014. Follow‐up was performed at 3, 6, and 12 months after ICU discharge.	IG: 193 patients underwent TCT. CG: 198 patients.	FIM, 6MWT, MRC, MCS and PCS, IES, BDI‐II, PFTs. ICU and hospital readmission. Number of specialty visits. Discharge disposition. Survival rate.	Patients who received TCT had extended MV, ICU, and hospital durations, as well as increased hospital death rates. They also experienced a greater daily care load, indicated by reduced FIM scores, along with worsened pulmonary function and 6MWT outcomes. The group with tracheostomy experienced a greater number of hospital readmissions and was less likely to be sent home.	AjD: PTSD. Connection Adj and QoL: The research revealed that individuals with TCT experienced an increased care burden and worse functional results, both of which can greatly impact their QoL. Although psychological assessments showed no significant differences, the results indicate that TCT is associated with a more complicated recovery after ICU, impacting both physical and emotional health. The difficulties in going back home and the physical restrictions also affect the patient's overall QoL.
Bozec et al. 2021 (Bozec et al. 2021) Location: tertiary care cancer center. Country: France.	To assess the long‐term QoL, functional, and psychological results in laryngectomized individuals who attained effective voice restoration through TEP.	Study design: cross sectional study. Size: 48 patients. Age: Age ≤ 75 years: 36 patients (75%); Age > 75 years: 12 patients (25%). Sex: Male: 36 patients (75%); Female: 12 patients (25%). Diagnosis: patients were identified with primary or recurrent squamous cell carcinoma of the larynx or hypopharynx and underwent total laryngectomy.	Patients had surgery performed from january 2009 to december 2018. The evaluation of QoL, functional performance, and psychological results was conducted in january 2020 (no less than one year post‐surgery).	This was a cross‐sectional study, rather than an interventional study featuring intervention and control groups. Every patient had experienced a total laryngectomy and had their voice restored using TEP.	EORTC; QLQ‐C30; QLQ‐H&N35; VHI‐10; DOSS, HADS.	The majority of patients indicated a good QoL and functional results, although fatigue and dyspnea were frequent issues. Problems with coughing, sensory functions, and speech were notable among head and neck symptoms. Although the majority of patients returned to a regular oral diet, around one‐third experienced psychological distress, which was a significant predictor of unfavourable voice and QoL results. Challenges related to social interaction, including withdrawal and isolation, were often noted, while issues with voice and speech continued to be prominent physical concerns.	AjD: anxiety and depression. Connection Adj and QoL: The research highlighted a significant connection between psychological suffering and low QoL as well as functional results. It emphasized the significance of psychological health in influencing social connections, with challenges such as withdrawal and isolation being prevalent. Psychological distress was identified as the main factor influencing poor QoL, even in instances of effective voice rehabilitation. This highlights that, even with physical advancements, mental health continues to be an important element affecting overall QoL.
Sanderson et al. 2016 (Freeman‐Sanderson et al. 2016) Location: in a large metropolitan ICU. Country: Australia.	To examine the effects of regaining voice on the quality of communication‐related life and overall health of TCT patients in the ICU.	Study design: observational study. Size: 22 patients who regained their voice during their time in the ICU. Age: Participants were adult patients, aged > 18 years. Sex: not specificated. Diagnosis: Patients had undergone a TCT and experienced voicelessness during MV.	Participants were recruited between august 2010 and 2014.	This was an observational study, not a controlled interventional study. All participants had undergone a TCT.	VASES, EQ‐5D.	The reintroduction of voice significantly improved patients' feeling of being heard and their general sense of happiness. Although no statistically significant enhancement in general health status was found based on the EQ‐5D, a trend indicating improvement was observed. The median differences observed in all eight VASES items examined supported the restoration of voice. These results indicate that recovering the ability to communicate positively affects specific areas of patients' wellness.	AjD: anxiety. Connection Adj and QoL: The research indicated that recovering the capacity to speak greatly enhanced communication‐related dimensions of QoL, favorably impacting psychological well‐being. Feeling understood and experiencing joy are vital for emotional adaptation in critically ill individuals. Reestablishing voice can alleviate negative feelings such as fear, sadness, anger, and frustration, ultimately improving overall QoL. Efficient communication enhances relationships with caregivers, which directly influences the patient's mental well‐being.
Sanderson et al. 2016 (Freeman‐Sanderson et al. 2016) Location: royal prince alfred hospital. Country: Australia.	To assess whether timely intervention through speech pathology services, such as cuff deflation and the application of an in‐line speaking valve during MV, would expedite the resumption of phonation in TCT patients when contrasted with standard care.	Study design: randomized controlled trial Size: 30 participants. Age: early IG: mean age 53 years; CG: mean age 67 years. Sex: early IG: 11 males (73%); CG: 6 males (40%). Diagnosis: participants had undergone a TCT and were MV, voiceless for at least 48 h.	Participants were recruited from august 2010 to september 2011 and october 2012 to august 2014.	IG: 15 participants. CG: 15 participants.	Time to phonation (ability to count from 1 to 10). Duration of TCT cannulation. Duration of MV. Length of stay in ICU. Length of stay in hospital. Time to oral intake. VASES; EQ‐5D.	The initial IG saw a markedly quicker resurgence to phonation than the CG. Nonetheless, there were no notable differences between the groups regarding tracheostomy cannulation duration, mechanical ventilation, ICU or hospital length of stay, or time until oral intake. Both groups experienced a similar and low frequency of adverse events. Although QoL indicators were more favorable for the early IG, the difference lacked statistical significance.	AjD: anxiety. Connection Adj and QoL: The research indicated that prompt reestablishment of communication, via phonation, might have a beneficial effect on the emotional well‐being of patients, as reflected in the VASES scores. Although the results lacked statistical significance, the trend supporting the early IG suggests possible enhancements in QoL related to communication. Communication is vital for a patient's mental well‐being, influencing their overall QoL. This emphasizes the significance of prompt action in improving both emotional and physical healing.

Abbreviations: Legend: quality of life (QoL), tracheostomy (TCT), intervention group (IG), control group (CG), quality of life was assessed using the university of Washington quality of life questionnaire version 4 (UWQOL v4), adjustment disorder (Adj), intensive cvare unit (ICU), mechanical ventilation (MV), renal replacement therapy (RRT), functional independence measure (FIM), 6‐minute walk test (6MWT), medical research council (MRC), short form (36) health survey (SF‐36), impact of event scale (IES), beck depression inventory‐II (BDI‐II), pulmonary function tests (PFTs), head and neck cancer (HNC), voice‐related quality of life (V‐RQOL), visual analog scale for anxiety (VAS‐A), neuromuscular diseases (NMD), chronic respiratory failure (CRF), home invasive mechanical ventilation (HIMV), amyotrophic lateral sclerosis (ALS), spinal muscular atrophy (SMA), duchenne muscular dystrophy (DMD), connor and davidson's resilience cale (CD‐RISC‐25), acceptance and action questionnaire‐II (AAQ‐II), state‐trait anxiety inventory (STAI), langer mindfulness scale (LMS), prolonged mechanical ventilation (PMV), long‐term acute care (LTAC), revised Edmonton Symptom Assessment System (r‐ESAS), geriatric depression scale (GDS), health‐related quality of life (HRQoL), acute respiratory distress syndrome (ARDS), short form‐12 (SF‐12), physical component summary (PCS‐12), mental component summary (MCS‐12), patient health questionnaire‐9 (PHQ‐9), post‐traumatic stress syndrome‐14 (PTSS‐14), obsessive‐compulsive disorder (OCD), post traumatic stress disorder (PTSD), hospital anxiety and depression scale (HADS), national health service (NHS), normalisation measure development (NoMAD), hospital‐community‐home (HCH), traumatic brain injury (TBI), glasgow coma scale (GCS), self‐anxiety scale (SAS), pulmonary function tests (PFTs), tracheoesophageal puncture (TEP), european organization for research and treatment of cancer (EORTC); core QoL questionnaire (QLQ‐C30); head and neck cancer QoL questionnaire (QLQ‐H&N35); voice handicap index 10 (VHI‐10); dysphagia outcome and severity scale (DOSS). visual analogue self‐esteem scale (VASES), euroQol‐5D questionnaire (EQ‐5D).

## Author Contributions

**Andrea Calderone**: conceptualization, validation, writing–original draft. **Francesco Corallo**: methodology, validation, resources, data curation, visualization. **Davide Cardile**: conceptualization, methodology, writing–original draft. **Carmela Rifici**: methodology, validation, resources, writing–review and editing. **Fabrizia Caminiti**: methodology, validation, resources, data curation, visualization. **Valeria Conti‐Nibali**: data curation, visualization. **Serena Filoni**: methodology, visualization, supervision. **Rocco Salvatore Calabrò**: resources, Funding acquisition, Project administration, Supervision.

## Conflicts of Interest

The authors declare no conflicts of interest.

### Peer Review

The peer review history for this article is available at https://publons.com/publon/10.1002/brb3.70517

## Data Availability

The data that support the findings of this study are not openly available due to reasons of sensitivity and are available from the corresponding author upon reasonable request.
